# Imaging Quality Control, Methodology Harmonization and Clinical Data Management in Stress Echo 2030

**DOI:** 10.3390/jcm10143020

**Published:** 2021-07-07

**Authors:** Ylenia Bartolacelli, Andrea Barbieri, Francesco Antonini-Canterin, Mauro Pepi, Ines Paola Monte, Giuseppe Trocino, Agata Barchitta, Alberto Cresti, Sofia Miceli, Licia Petrella, Frank Benedetto, Concetta Zito, Giovanni Benfari, Francesca Bursi, Alessandro Malagoli, Francesca Mantovani, Quirino Ciampi, Angela Zagatina, Eszter Dalma Palinkas, Attila Palinkas, Szilvia Rostasne Toth, Karina Wierzbowska-Drabik, Ana Djordievic-Dikic, Patricia A. Pellikka, Eugenio Picano, on behalf of the Stress Echo 2030 Study Group of the Italian Society of Echocardiography and Cardiovascular Imaging

**Affiliations:** 1 Pediatric Cardiology and Adult Congenital Heart Disease Program, Department of Cardio-Thoracic and Vascular Medicine, IRCCS Azienda Ospedaliero-Universitaria di Bologna, 40138 Bologna, Italy; 2 Department of Diagnostics, Division of Cardiology, Clinical and Public Health Medicine, Policlinico University Hospital of Modena, 41125 Modena, Italy; barbieriandrea65@gmail.com; 3 Cardiac Prevention and Rehabilitation Unit, Highly Specialized Rehabilitation Hospital Motta di Livenza (TV), Treviso and Italian Society of Echocardiography and Cardiovascular Imaging, 31045 Motta di Livenza, Italy; antonini.canterin@gmail.com; 4 Monzino Cardiology Center, IRCCS, 20138 Milano, Italy; Mauro.Pepi@cardiologicomonzino.it; 5 Division of Cardiology, AOU Policlinic, University of Catania, 95123 Catania, Italy; inemonte@gmail.com; 6 Division of Cardiology, San Gerardo Hospital-ASST Monza, 20900 Monza, Italy; g.trocino@gmail.com; 7 Division of Emergency Medicine, S. Antonio Hospital, AO Padova, 35127 Padova, Italy; agathabarchitta@gmail.com; 8 Division of Cardiology, Dip. CardioNeuroVascolare Aslsudest Toscana, Hospital of Grosseto, 58100 Grosseto, Italy; alcresti@gmail.com; 9 Division of Geriatrics, AOU Mater-Domini, 88100 Catanzaro, Italy; sofymiceli@libero.it; 10 Division of Cardiology, Mazzini Hospital, 64100 Teramo, Italy; petrella.licia@gmail.com; 11 Division of Cardiology, G.O.M. Bianchi Melacrino Morelli, 89133 Reggio Calabria, Italy; frankbenedetto@gmail.com; 12 Department of Clinical and Experimental Medicine-Section of Cardiology, G. Martino General Hospital, University of Messina, 98124 Messina, Italy; tittizito@libero.it; 13 Section of Cardiology, Department of Medicine, University of Verona, 37126 Verona, Italy; givanni.benfari@gmail.com; 14 Division of Cardiology, Department of Health Sciences, San Paolo Hospital, ASST Santi Paolo and Carlo, University of Milan, 20142 Milano, Italy; francescabursi@gmail.com; 15 Division of Cardiology, Nephro-Cardiovascular Department, Baggiovara Hospital, 41126 Baggiovara, Italy; ale.malagoli@gmail.com; 16 Division of Cardiology, Azienda USL-IRCCS di Reggio Emilia, 42123 Reggio Emilia, Italy; francy_manto@hotmail.com; 17 Division of Cardiology, Fatebenefratelli Hospital, 82100 Benevento, Italy; qciampi@gmail.com; 18 Cardiology Department, Saint Petersburg State University Clinic, Saint Petersburg State University, 199034 St. Petersburg, Russia; zag_angel@yahoo.com; 19 Department of Internal Medicine, Division of Non-Invasive Cardiology, Albert Szent-Gyorgyi Clinical Center, Doctoral School of Clinical Medicine, University of Szeged, 6725 Szeged, Hungary; palinkaseszti@hotmail.com; 20 Internal Medicine Department, Elisabeth Hospital, 6800 Hodmezovasarhely, Hungary; palinkasa@hotmail.com (A.P.); toth.szilvi790122@gmail.com (S.R.T.); 21 Chair of Cardiology, Bieganski Hospital, Medical University, 90-419 Lodz, Poland; wierzbowska@ptkardio.pl; 22 Clinical Center of Serbia, 11000 Belgrade, Serbia; skali.ana7@gmail.com; 23 Department of Cardiovascular Disease, Mayo Clinic, Rochester, MN 55905, USA; pellikka.patricia@mayo.edu; 24 Biomedicine Department, CNR, Institute of Clinical Physiology, 56124 Pisa, Italy; picano@ifc.cnr.it

**Keywords:** stress echo, redcap, methodology

## Abstract

Stress echo (SE) 2030 study is an international, prospective, multicenter cohort study that will include >10,000 patients from ≥20 centers from ≥10 countries. It represents the logical and chronological continuation of the SE 2020 study, which developed, validated, and disseminated the “ABCDE protocol” of SE, more suitable than conventional SE to describe the complex vulnerabilities of the contemporary patient within and beyond coronary artery disease. SE2030 was started with a recruitment plan from 2021 to 2025 (and follow-up to 2030) with 12 subprojects (ranging from coronary artery disease to valvular and post-COVID-19 patients). With these features, the study poses particular challenges on quality control assurance, methodological harmonization, and data management. One of the significant upgrades of SE2030 compared to SE2020 was developing and implementing a Research Electronic Data Capture (REDCap)-based infrastructure for interactive and entirely web-based data management to integrate and optimize reproducible clinical research data. The purposes of our paper were: first, to describe the methodology used for quality control of imaging data, and second, to present the informatic infrastructure developed on RedCap platform for data entry, storage, and management in a large-scale multicenter study.

## 1. Introduction

Stress echo (SE) 2030 study is an international, prospective, multicenter cohort study aiming to include >10,000 patients from ≥20 centers from ≥10 countries [1]. The study is the logical and chronological continuation of the SE 2020 study, which recruited 10,000 patients from 2016 to 2020 [2]. SE 2020 proposed, developed, validated, and disseminated a new protocol named ABCDE of SE, which is more comprehensive than the conventional protocol based only on regional wall motion abnormalities which has been utilized for 40 years from 1980 to 2020 [3]. In the newly revised format, SE’s ABCDE protocol is more versatile and more suitable to describe the complex vulnerabilities of the contemporary patient within and beyond coronary artery disease [4,5].

The new state-of-the art cardiac functional testing with stress echo ABCDE protocol employs and integrates five different steps: step A for regional wall motion abnormalities; step B for B-lines obtained with four-site simplified scan and lung ultrasound; step C for contractile reserve with volumetric echocardiography; step D for Doppler-based assessment of coronary flow reserve in mid-distal left anterior descending coronary artery; step E for EKG-based heart rate reserve. The five steps focus on different pathophysiological targets: epicardial coronary stenosis for step A; alveolar–capillary membrane and diastolic function for step B; left ventricular fibrosis or necrosis with step C; coronary microvascular dysfunction for step D; and sympathetic reserve for step E. The five parameters converge conceptually, logistically, and methodologically in ABCDE stress echo, and each one provides independent and incremental prognostic value since they explore different aspects of patient vulnerability. The overall imaging time is 30′ or less (with 3′ minutes of extra-imaging time for Step D and 1 min for step B). The extra-analysis time compared with standard stress echo based on regional wall motion abnormalities is <5′ (<3 min for volume calculation in step C, <1′ for step B, <1′ for step D and step E). The technicalities are very simple for all steps and parts of the standard training, except step D which requires coronary flow imaging of left anterior descending artery and dedicated training. Steps A and C are obtained in the very same images and projections employed for conventional step A (four-chamber and two-chamber apical views). Step B requires a one-minute lung ultrasound scan, usually in the early recovery phase (since pulmonary congestion does not disappear immediately upon stress cessation). Step E is imaging-independent and requires only one lead on the echo monitor.

All centers will enroll patients with the preferred stress modality according to the local laboratory policy and experience, patients’ characteristics, and available expertise or technology. For instance, some laboratories do not have a semi-supine bike, for others adenosine is too expensive, in some countries, dipyridamole is not commercially available. The study design does not interfere with clinical practice and accepts all stress modalities performed according to the standardized protocol of guidelines and recommendations. Abnormal cut-off values are common to all stresses for step A (wall motion abnormalities in two contiguous segments), step B (≥2 increase in B-lines with a four-site simplified scan) and step D (coronary flow velocity reserve < 2.0). Abnormal criteria are stress-specific for step C and step E, since exercise and dobutamine are stronger inotropic and chronotropic stresses than vasodilators. The abnormality cut-off for step C based on left ventricular contractile reserve from force (systolic blood pressure/end-systolic volume) is <2.0 for exercise and dobutamine, and <1.1 for dipyridamole and adenosine. The abnormality cut-off for step E is <1.80 for exercise and dobutamine, and <1.22 for dipyridamole and adenosine

On the ABCDE-SE methodological platform, SE2030 was started with a recruitment plan from 2021 to 2025 (and follow-up to 2030) with 12 subprojects (ranging from coronary artery disease to valvular and post-COVID-19 patients). With these features, the study offers unique scientific opportunities and poses particular challenges on quality control assurance, methodological harmonization, and data management.

It is well known that the need to capture, process, interpret and share clinical research data with multiple institutions is a significant challenge [6]. Sites enter data manually, which requires time for clinical workers and raises the risk of data errors [7,8]. Computerized methods are needed for collecting data and maintaining health records. However, they are also not free from recognized sources of error, and few guidelines are available to help accomplish this task [9]. The in-house software development fits local informatic technology environments and workflows but takes time and effort to extend beyond the original domain.

One of the significant upgrades of SE2030 compared to SE2020 was developing and implementing a Research Electronic Data Capture (REDCap)-based infrastructure for interactive and entirely web-based data management. The first REDCap project was launched at Vanderbilt University in August 2004. This clinical research software application has evolved beyond its single institutional use case to be shared with organizations worldwide. In recent years, the number of U.S. and international REDCap partners has increased exponentially [10,11]. The REDCap Consortium now has 4889 active partners in 141 countries. REDCap software has generated over 1,136,000 projects from over 1,647,000 users; 12,956 journal articles cite REDCap [12].

This paper illustrates the methodology used in SE2030 for quality control of SE data and develops REDCap’s SE2030 infrastructure for data submission, storage, and management, intending to integrate and optimize repeatable clinical data research.

The future of high-quality imaging research is to build image and data bank of large dimensions and representative of real-world situations, but the best way to reconcile quality control of imaging data, with resource economy and integration of data remains a challenge. The purpose of the present work is to present our proposed approach to integrate and optimize clinical data research applied to a specific imaging rest and stress echocardiographic study which poses unique challenges of data integration and unique opportunities of data mining.

## 2. Quality Control of Cardiac Ultrasound and Stress Echo Data

Imaging with cardiac ultrasound is a key component of contemporary cardiological research, but the value of the obtained information is highly dependent on the quality of imaging, robustness of data handling and storage, and strategy of analysis. A possible approach when dealing with images is to develop quality control through a core laboratory reading all studies. This means that dedicated economic, human, and technological resources are needed [13,14]. The core SE lab approach was not compatible with the aims, available resources, and research strategy of SE2030.

The strategy of obtaining data directly from peripheral centers is characterized by substantially lower cost and complexity. Yet, we know that some techniques do not tolerate improvisation. Cardiac ultrasound in general, and especially SE, have a recognized limitation in dependence on the operator. Different operators have different levels of expertise, consistency, and accuracy. Our strategy consisted of the upstream evaluation of readers before entering the data bank. This strategy was used for 30 years of different waves of SE multicenter studies, but it is now web-based and completely digital with further sparing of time and resources for all participants. Upstream control and then direct enrollment of data from peripheral centers is also a prerequisite for an effectiveness study that intends to assess the technique’s performance outside highly specialized academic centers. It also gives the same importance to the academic center and the peripheral recruiting center, which often has very little to learn from the coordinating echocardiographic laboratory. The result is creation of a diffuse sparse, bottom-up knowledge network rather than a pyramidal top-down geometry with the coordinating core echocardiographic lab on top.

**Figure 1:**
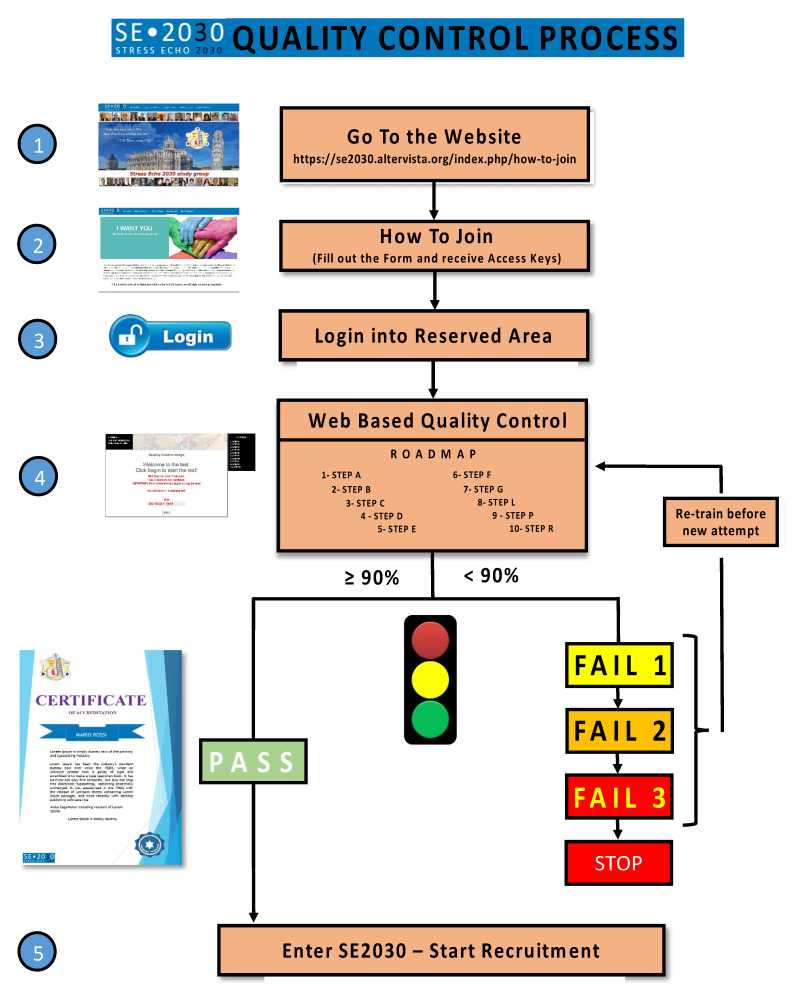
Stress echo 2030: road to accreditation.

The readers’ certification by official societies such as the American Society of Echocardiography or European Association of Cardiovascular Imaging and a high-volume laboratory are prerequisites. The second necessary criterion is the specific quality control for each of the essential steps of a comprehensive SE study, from conventional step A (regional wall motion assessment) to a more advanced step B (B-lines), step C (volumetric echocardiography), step D (Doppler-based assessment of coronary flow velocity reserve in left anterior descending coronary artery) and step E (imaging independent heart rate reserve). Other credentialing steps for specific subprojects include the assessment of mitral insufficiency (step F), left atrial volume (step L), pulmonary artery systolic pressures (from tricuspid regurgitant jet velocity, step P), etc. For each step, a dedicated web-based module is used to harmonize methodology and share reading criteria (Figure 1). The prospective participant will enter a dedicated website with different steps to read selected 20 cases per step. The requisite for certification is the concordance equal or greater than 90% compared with the gold standard represented by the unanimous reading of two senior and experienced observers [15]. The website also clarifies the reading criteria, the terminology used, the methodology of the scan, and the rationale of the employed approach. In this way, the reading is reasonably harmonized, and all readers are quality controlled prior to having access to entering the database.

In a specific sub-study of SE2030, the reading of left ventricular volumes and wall motion will be compared head-to-head with artificial intelligence-based evaluation. This will eventually also allow an evaluation of how much each center may deviate from the gold standard reading of an operator-independent assessment.

The principal investigator will take the responsibility of quality control of candidate recruiting centers according to the criteria and modalities already adopted for stress echo 2020.

## 3. Data Quality Assurance

In general, REDCap is easy to use for research study participants removing the need for manual entry of data, using free or inexpensive and readily available tools, lending itself well to eventual sharing of open source material. No structured programming, networking, or database expertise is required because, on the REDCap consortium website, training videos and support tools are available to familiarize users with the platform [16].

The evaluation’s visual interface is appealing and intuitive, integrating web-based usability best practices with answer types in a way that most users are familiar with. Standardized data collection software built in REDCap, followed by a comprehensive standard operating procedure guide, ensures uniform data collection [17].

Although it is the investigators’ responsibility to ensure that all data are entered fully and correctly in the respective database during the study, data entry fields are provided with input instructions. They are pre-programmed to accept values only within a possible range. A user-friendly and sustainable platform is generated using uniform variable definitions and automated verification of data and set ranges for date and numeric fields. The user will be alerted whenever entered data violates specified limits, facilitating data validation. Data consistency issues such as incorrect data form, out-of-range values, and outliers for numerical fields may be identified using the data quality module. Additionally, it is possible to introduce pre-defined rules and advanced features such as branching logic and calculated fields, making it easier to decide if a particular data value might be inconsistent, which is very necessary since there are many fields and many records in SE2030. Therefore, the implementation of all these REDCap tools decreases the probability of data entry errors and the risk of research personnel transcription errors.

**Figure 2:**
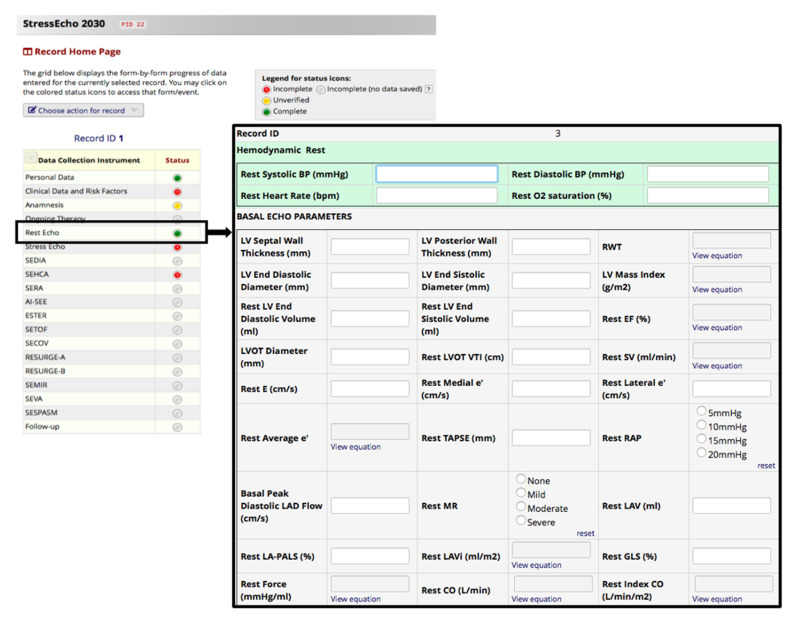
Record status dashboard. eCRF pages and REDCap functional modules are accessible to end-users by clicking links on each project’s right-side application menu.

Data from centers that are part of the SE2030 working group are progressively acquired during routine clinical care. Indeed, it is helpful for the researchers that the eCRFs are kept current to reflect participant status at each phase during the study. For this purpose, in REDCap, each data collection instrument’s status is recorded (Figure 2): the default setting of ‘incomplete’ can be upgraded to ‘unverified’ when data are entered but not yet verified and to ‘complete’ when an experienced researcher has carried out data verification (rights are pre-specified in the user account of the researcher).

During the input process, all SE2030 qualified medical staff members will work together on data extraction, and the information will be cross-checked. Each subproject coordinator will check all medical records of the first 20 patients to ensure that standard operating procedures are adherent, including data collection and entry. Spot checks will also be performed every two months by a statistician. Five or more input errors per 100 will trigger a full-scale analysis of all clinical data or re-enter and reassess them.

For quality assurance, database support, and avoidance of duplicate entries, only SE 2030 research coordinators will have access to the complete database to manage data access and entry by local researchers at each site.

## 4. Protection of Human Subjects

The health-related personal data provided by REDCap are strictly confidential, and disclosure to third parties is prohibited. The administration and subsequent coding of in-person SE2020 data were among the most labor-intensive processes throughout the research center’s studies before REDCap implementation. In SE2030, the data and query management, monitoring, reporting, and coding will be collected following the Good Clinical Practice guidelines on internet-based secure data provided by Vanderbilt University [18].

REDCap is a protected web application that enhances data security by multiple layers of encryption and common-sense security practices. Database access will be limited to unique data access groups corresponding to the participating centers to ensure that researchers can access data from patients known in their centers. Only the investigator or other designated persons will be able to conduct corrections in the eCRFs. Patients cannot be identified in the eCRFs by name since an appropriate coded identification (e.g., Participant Number) will have to be used to recognize any authorized user who can carry out data entries and eCRF changes. The code will be protected against unauthorized access and stored appropriately by the study coordinator assigned to the study.

In the case of corrections, the original data entries will be archived and visible on the system. For all data entries and revisions, the date, time of day, and the person making the entries will be generated automatically.

## 5. Database Management

REDCap has limited staff requirements (one support person can manage all projects of SE2030 easily) but requires a typical web infrastructure [8]. Therefore, SE2030 has entered into a partnership with the Italian Society of Echocardiography and Cardiovascular Imaging (SIECVI), providing informatics technology support.

**Figure 3:**
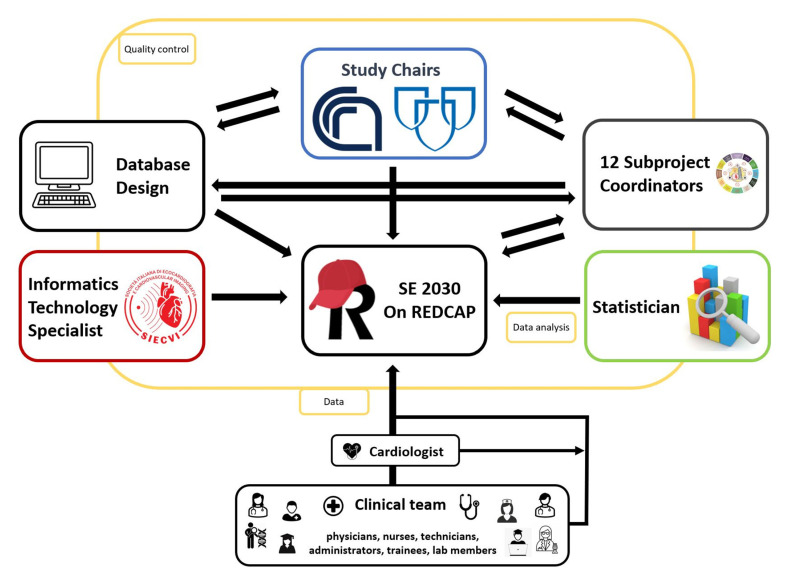
Schematic representation of the workflow methodology for building a REDCap database for the SE2030 study: data access, logistics, and sharing.

An informatics technology professional was assigned to assist with project programming to ensure that the database will be consistent with standard operating procedures. Study data were collected and managed using REDCap electronic data capture tools. The study data bank will be stored and jointly by Mayo Clinic and SIECVI. To handle SE2030, the study chairs, subproject coordinators, physicians, an information technology expert, and a statistician will work together. The study chairs will manage all the database-related staff tasks (Figure 3).

Based on conversations conducted with the research team, the database layout has been developed. Using REDCap, eCRFs were generated by the information technology specialist and checked by the statistician. Of note, data collections in Microsoft Excel and Microsoft access can be easily converted to REDCap. Data can be downloaded to Excel, PDF, SAS, SPSS, and Stata.

The implementation steps include (1) completion and submission of all required information on the main project settings, (2) creation of data collection tools by defining data variables and their properties, (3) preview and checking of the data entry screens to ensure sufficient data set yields for expected statistical analyses, (4) permissions and user privileges configuration for research participant permissions, and (5) the transfer of the project from conception to production to ensure data quality and credibility after the actual clinical data collection has begun.

The “burden” of sharing was not restricted to the initial configuration. When new features have been introduced, bugs have been exposed and corrected by the project manager.

The study chairs and the statistician supervise all data activities to ensure quality control. The “front end” meets best practices for user usability across different devices such as phones, tablets, and computers, thus reducing the pressure on the research team’s participants to make self-reports and ideally leading to the retention of participants.

## 6. Tips and Tricks for Safe Use of REDCap

There are three tightly interconnected steps for the safe use of REDCap data entry, database maintenance, and data analysis. In SE2030, data entry is the responsibility of the cardiologist accredited for reading in the peripheral center. The cardiologist may decide to enter the data directly in REDCap or supervise data entry by a person (usually a junior colleague, a nurse, or a technician) under his/her direct supervision. Database maintenance is the cardiologist’s responsibility, skilled and trained in data management, who has built the database according to the clinical team’s directive and will remodel based on the clinical end-user’s input. Data analysis is the statistical consultant’s responsibility, who was also responsible for the statistical plan of the study protocol. The clinical team is involved in all three steps and must effectively interface with all key players to reach the study goals. In this complex teamwork, there are at least three possible mistakes.

The first mistake to avoid is considering the three key players as entirely independent. They must interact and communicate efficiently for adapting and reshaping the program according to the diverging needs of the end-user (“less is more”, less variables mean less time required to input the study) and the clinical team (“more is more”, many variables may prove interesting at the end). In the middle of these opposite forces, the data management team should ensure simplicity, feasibility, and flexibility—which is more easily said than done.

A second possible mistake is to take as final and untouchable the clinical team’s proposal (usually experienced and established scientists). The real final judge is the clinical end-user. In SE2030, there were countless rounds of changes, corrections, modifications, adjustments, and adaptations suggested by the clinical end-users who offered their feedback on the clinical coordinating team and data management team. The final version is substantially different and unquestionably better than the one initially prepared by the clinical team. The most common identified problems were duplication of data (repeated on the main page of the study and for specific subprojects) and often the inclusion of too many redundant data of questionable or marginal relevance (“just in case” variables). Compared to the initial draft inflated of variables, there was a significant reduction (about 20%) in included items. The clinical team’s interaction with the end-users and data management team taught everyone that any marginally useful (or likely useless) variable is also a potential source of error, a waste of time, and a human and economic cost. On the other side, sometimes, the clinical team did not provide the level of specificity required by the data management team, and some items had to be expanded upon and detailed.

The third possible mistake is also the most common. The program is simple but—especially for first-time users—not so simple that you can skip education, training, communication, and verification. Redcap is intuitive but has a learning curve. It is user-friendly, but “*it can open the door for error/disaster without proper education, due diligence, training, and communication: test, test, and test again!*” [19,20]

## 7. Conclusions

These findings highlight the advantages of using web-based quality control and credentialing of prospective readers and computerized data collection and management system (Table 1). Web-based computerized data collection and management save time in all phases of research studies [9].

SE2030 was designed and implemented on the REDCap platform, which provides an intuitive interface for validated data entry, and audit trails for tracking the history of data entry and revision, integrated calendar scheduling, and ad hoc reporting tools. We are confident that the web-based optimization with REDCap, using automation practices for data flow, will create a SE2030 database that can serve as a simple and accurate data collection, storage, and management tool.

## Figures and Tables

**Table 1:** Summary of the potential advantages of a web-based computerized data collection and management system. Adapted from https://www.ualberta.ca/medicine/media-librSary/resources/medit/comparison---excel-access-redcap--2015-09-03.pdf (accessed on 1 March 2021).

	Excel^®^	Access^®^	REDCap™
Secure location—health information resides on a secure server	Only if the file resides on a secure server, not on a PC	Only if the file resides on a secure server, not on a PC	Yes. REDCap resides on the FoMD’s secure server and is accessed via the internet
Authentication—logins and passwords	No	No	Yes
Authorization—role-based security	The file owner can set access to data	Access to data can be set by the file owner with the installation of MS Information Rights Manager PlugIn for Office 2007	User accounts are controlled by the REDCap Administrator. Access to individual databases is controlled by the owner of the database
Data validation	No	Yes (Requires programming effort)	Yes. (Automatically configured, no programming required. Includes sophisticated data quality tools)
Vulnerabilities	Easy to corrupt or lose data	Easy to corrupt data if not programmed correctly	Limited flexibility in form design
Layout in form view for easy data entry	No	Yes	Yes
Multiple user access to data	No	Record-level locking	Yes, but no locking; last save overwrites previous save
Unique identifiers	No	No	Yes
File size limitations	255 columns	Very large	Scalable, based on server size
Exporting data	CSV	ODBC compliant	CSV/Excel, SAS, SPSS, Stata, R (export tool creates syntax files)
Sort and filter data	Moderate	Strong	Very Simple
Secure file transmission	No	No	Yes, using Send-It feature

## Data Availability

Not applicable.

## References

[1] Picano E., Zagatina A., Wierzbowska-Drabik K., Daros C.B., D’Andrea A., Ciampi Q. (2020). Sustainability and Versatility of the ABCDE Protocol for Stress Echocardiography. J. Clin. Med..

[2] Picano E., Ciampi Q., Citro R., D’Andrea A., Scali M.C., Cortigiani L., Olivotto I., Mori F., Galderisi M., Costantino M.F. (2017). Stress echo 2020: The international stress echo study in ischemic and non-ischemic heart disease. Cardiovasc. Ultrasound.

[3] Pellikka P.A., Arruda-Olson A., Chaudhry F.A., Chen M.H., Marshall J.E., Porter T.R., Sawada S.G. (2020). Guidelines for Performance, Interpretation, and Application of Stress Echocardiography in Ischemic Heart Disease: From the American Society of Echocardiography. J. Am. Soc. Echocardiogr..

[4] Scali M.C., Zagatina A., Ciampi Q., Cortigiani L., D’Andrea A., Daros C.B., Zhuravskaya N., Kasprzak J.D., Wierzbowska-Drabik K., Pretto J.L.D.C.E.S. (2020). Lung Ultrasound and Pulmonary Congestion During Stress Echocardiography. JACC Cardiovasc. Imaging.

[5] Ciampi Q., Zagatina A., Cortigiani L., Gaibazzi N., Daros C.B., Zhuravskaya N., Wierzbowska-Drabik K., Kasprzak J.D., Pretto J.L.D.C.E.S., D’Andrea A. (2019). Functional, Anatomical, and Prognostic Correlates of Coronary Flow Velocity Reserve During Stress Echocardiography. J. Am. Coll. Cardiol..

[6] Sung N.S., Crowley J.W.F., Genel M., Salber P., Sandy M.L., Sherwood L.M., Johnson S., Catanese V., Tilson H., Getz K. (2003). Central Challenges Facing the National Clinical Research Enterprise. JAMA.

[7] Cummings J., Masten J. (1994). Customized dual data entry for computerized data analysis. Qual. Assur..

[8] Reynolds-Haertle R.A., McBride R. (1992). Single vs. Double data entry in CAST. Control. Clin. Trials.

[9] Rosenberg R.N. (2003). Translating biomedical research to the bedside: A national crisis and a call to action. JAMA.

[10] Harris P.A., Taylor R., Minor B.L., Elliott V., Fernandez M., O’Neal L., McLeod L., Delacqua G., Delacqua F., Kirby J. (2019). The REDCap consortium: Building an international community of software platform partners. J. Biomed. Inform..

[11] Harris P.A., Taylor R., Thielke R., Payne J., Gonzalez N., Conde J.G. (2009). Research electronic data capture (REDCap)—A metadata-driven methodology and workflow process for providing translational research informatics support. J. Biomed. Inform..

[12] US Department of Health and Human Services (2013). Modifications to the HIPAA Privacy, Security, Enforcement, and Breach Notification rules under the Health Information Technology for Economic and Clinical Health Act and the Genetic Information Nondiscrimination Act; other modifications to the HIPAA rules. Federal Register.

[13] Douglas P.S., DeCara J.M., Devereux R.B., Duckworth S., Gardin J.M., Jaber W.A., Morehead A.J., Oh J.K., Picard M., Solomon S.D. (2009). Echocardiographic Imaging in Clinical Trials: American Society of Echocardiography Standards for Echocardiography Core Laboratories. J. Am. Soc. Echocardiogr..

[14] Galderisi M., Henein M.Y., D’Hooge J., Sicari R., Badano L., Zamorano J.L., Roelandt J.R.T.C., European Association of Echocardiography (2011). Recommendations of the European Association of Echocardiography How to use echo-Doppler in clinical trials: Different modalities for different purposes. Eur. J. Echocardiogr..

[15] Ciampi Q., Picano E., Paterni M., Daros C.B., Simova I., Pretto J.L.D.C.E.S., Scali M.C., Gaibazzi N., Severino S., Djordjevic-Dikic A. (2017). Quality control of regional wall motion analysis in stress Echo 2020. Int. J. Cardiol..

[16] Klipin M., Mare I., Hazelhurst S., Kramer B. (2014). The process of installing REDCap, a web based database supporting biomedical research: The first year. Appl. Clin. Inform..

[17] Weber B.A., Yarandi H., Rowe M.A., Weber J.P. (2005). A comparison study: Paper-based versus web-based data collection and management. Appl. Nurs. Res..

[18] https://www.project-redcap.org/.

[19] https://redcapdemo.vanderbilt.edu/.

[20] Ciolino J.D. Clinical Database Management Using REDCap: Tips and Tricks for Efficient Data Capture. Northwestern Medicine. 12 February 2019. https://www.feinberg.northwestern.edu/sites/nudacc/docs/ciolino_overview_redcap.pdf.

